# Clinical application of a microfluidic chip for immunocapture and quantification of circulating exosomes to assist breast cancer diagnosis and molecular classification

**DOI:** 10.1371/journal.pone.0175050

**Published:** 2017-04-03

**Authors:** Shimeng Fang, Hongzhu Tian, Xiancheng Li, Dong Jin, Xiaojie Li, Jing Kong, Chun Yang, Xuesong Yang, Yao Lu, Yong Luo, Bingcheng Lin, Weidong Niu, Tingjiao Liu

**Affiliations:** 1 College of Stomatology, Dalian Medical University, Dalian, China; 2 Department of Urology, the Second Affiliated Hospital, Dalian Medical University, Dalian, China; 3 Department of Nuclear Medicine, the First Affiliated Hospital, Dalian Medical University, Dalian, China; 4 Department of Biochemistry and Molecular Biology, Liaoning Provincial Core Lab of Glycobiology and Glycoengineering, Dalian Medical University, Dalian, China; 5 Department of Biotechnology, Dalian Institute of Chemical Physics, Chinese Academy of Sciences, Dalian, China; 6 State Key Laboratory of Fine Chemicals, Department of Chemical Engineering, Dalian University of Technology, Dalian, China; University of South Alabama Mitchell Cancer Institute, UNITED STATES

## Abstract

Increasing attention has been attracted by exosomes in blood-based diagnosis because cancer cells release more exosomes in serum than normal cells and these exosomes overexpress a certain number of cancer-related biomarkers. However, capture and biomarker analysis of exosomes for clinical application are technically challenging. In this study, we developed a microfluidic chip for immunocapture and quantification of circulating exosomes from small sample volume and applied this device in clinical study. Circulating EpCAM-positive exosomes were measured in 6 cases breast cancer patients and 3 healthy controls to assist diagnosis. A significant increase in the EpCAM-positive exosome level in these patients was detected, compared to healthy controls. Furthermore, we quantified circulating HER2-positive exosomes in 19 cases of breast cancer patients for molecular classification. We demonstrated that the exosomal HER2 expression levels were almost consistent with that in tumor tissues assessed by immunohistochemical staining. The microfluidic chip might provide a new platform to assist breast cancer diagnosis and molecular classification.

## Introduction

Exosomes are small cell-derived vesicles of 40–100 nm that are present in many and perhaps all biological fluids [1–3]. They carry various molecular components of their cell of origin, including lipids, proteins, mRNAs, microRNAs (miRNAs), long non-coding RNA (lncRNA), and even DNA [4–6]. Exosomes can merge with and release their contents into recipient cells, thus transfer their cargo from one cell to another. Increasing evidence has demonstrated that exosomes play an important role in cell-to-cell communication and influence both physiological and pathological processes [4, 7–9].

Cancer cells release more exosomes in serum than normal cells and these exosomes overexpress a certain number of cancer-related biomarkers [10–12]. Recently, exosomes draw much attention as a promising biomarker for cancer screening, diagnosis and prognosis because they are easily accessible and capable of representing their parental cells [13–15]. The common methods for isolation of exosomes mainly depend on non-specific physicochemical properties such as particlesize, density and solubility. Ultracentrifugation is the most commonly used method for concentration of exosomes. It needs the centrifuge speed at least over 100,000 *G* that requires special laboratory machine operated by specialists [16]. Commercialized exosome isolation Kits have been developed as well. However, all of these methods are relatively expensive, large sample required, and cannot separate exosomes from other extracellular vesicles (EVs) thoroughly.

Microfluidic-based exosome manipulation techniques have been developed since 2010 and proved their advantages, such as small sample volume, low cost, product purity, and short operation time [17, 18]. The techniques developed so far for microfluidic-based exosomal concentration can be classified into two categories, including sized-based and biomarker-based approaches [19]. According to the different size of exosomes from other EVs, several microlfuidic devices were developed. Davies R. et al. presented a microfluidic filtration system to isolate vesicles from whole blood samples [20]. Direct current electrophoresis was employed as an alternative driving force to propel particles across the filter and increase the separation efficiency of vesicles from proteins. Wang Z et al. fabricated a microfluidic device consisting of ciliated micropillars, forming a porous silicon nanowire-on-micropillar structure that preferentially trapped exosome-like lipid vesicles [21]. Several proteins are recognized as specific exosomal markers that are commonly used to distinguish exosomes from other EVs, such as CD63, CD81, and MHC I. Thus immuno-chips were constructed to capture exosomes based on the expression of different surface biomarkers [22–25]. Anti-CD63 antibody was commonly chosen for exosomal capture from all cell origins because of its high expression levels. In addition, antibodies targeting specific cancer antigens could be used to detect cancer specific exosomes for cancer diagnosis and monitoring treatment response. Zhao Z. et al. reported a microfluidic chip for blood-based diagnosis of ovarian cancer by multiplexed measurement of three exosomal tumor markers (CA-125, EpCAM, CD24) [24]. Immuno-capture technique is considered as the only ones, so far developed, which can be directed towards the capture of a pure exosome population. Other methods relying on physical properties (size, density, surface charge) lead to higher percentages of contaminants (similar EVs with different origin and proteins).

In this study, we developed a microfluidic device that enables on-chip immunocapture exosomes from both cell culture medium and patient plasma. Subsequently, exosomal expression of tumor specific antigens could be detected and quantified using immunofluorencent staining. We applied this microfluidic technology to study breast cancer-derived exosomes. It was demonstrated that the amount of EpCAM-positive exosomes showed significantly higher in the plasma of breast cancer patients than that in healthy controls. HER2 is a therapeutic target in breast cancer and current clinical assessment of its expression relies on immunohistochemical staining of tumor tissues [26, 27]. We proved that exosomal expression of HER2 in the plasma of breast cancer patients was almost consistent with that in tumor tissues.

## Materials and methods

### Chip fabrication

The microfluidic device is composed of a glass substrate and a polydimethylsiloxane (PDMS) membrane. The PDMS membrane was fabricated by repeated molding of the master, which was prepared by spin coating a 100-μm-thick layer of SU8-3035 negative photoresist (Microchem Corp., Newton, CA, USA) onto a glass wafer and patterned by photolithography. The Sylgard 184 PDMS base and curing agent (Dow Corning, Midland, MI, USA) were mixed thoroughly (10:1 by mass), poured onto the mold and cured in an oven at 80℃ for 1 hour. After cooling, the PDMS membrane was peeled from the master and trimmed to size. Inlet and outlet holes were punched out of the PDMS. Then the PDMS membrane was bonded to a glass slide after plasma treatments.

### Cell culture and preparation of conditioned cell culture medium (CM)

Three types of human breast carcinoma cell line, including MCF7, MDA-MB-231 (M231) and SK-BR-3, were used in this study. Primary human normal fibroblasts (NF) were isolated as described previously and used as a control [28]. MCF7 and NF were cultured in Dulbecco's Modified Eagle Medium: Nutrient Mixture F-12 (Hyclone, Logan, UT, USA). M231 was cultured in L-15 (Macgene, Beijing, China). SK-BR-3 was cultured in McCoy’s 5A medium (Macgene). All of the cell culture media were supplemented with 10% fetal bovine serum (Hyclone), 100 U/mL penicillin, and 100 U/mL streptomycin. All cells were cultured at 37°C with 5% CO_2_ and 95% relative humidity. CM was collected from serum-free medium cultured on each cell line for 48 h, and centrifuged at 2000 rpm for 15 min to delete cell debris for exosome capture. All of the samples were stored at −80°C until use.

### Plasma sample preparation

All studies involving human materials were approved by the Research Ethics Committee, Dalian Medical University, China. Participants provide their written informed consent to participate in this study. The ethics committees/IRBs approve this consent procedure. All samples were obtained from the Second Affiliated Hospital, Dalian Medical University. Human blood samples were collected from healthy donors and breast cancer patients. Samples were centrifuged for 30 min at 5000 *G*. Then the top layer of plasma was carefully collected and stored at −80°C until use.

### Transmission electron microscopy (TEM) imaging

TEM imaging was performed to characterize the size and morphology of exosomes. A drop of purified exosome sample was placed onto 200 mesh copper grids, and allowed to absorb for 15 min. Grids were dried by filter paper and exosomes were then counterstained for 5 min with phosphato-tungstic acid, then imaged by a TEM (GEM-2000EX, Japan Electronics, Japan).

### Immunocaptureand quantification of exosomes on the microfluidic chip

To capture exosomes, CD 63 antibody (Abcam, Cambridge, UK) was conjuncted with magnetic nanoparticles (Mag-CD63; Wuhan Jiayuan Quantum Dots Corporation, Wuhan, China). The final concentration of Mag-CD63 was 1mg/ mL and the typical binding capacity was tested to be about ~10 μg CD63 antibody per 1 mg of nanopartilces. Mag-CD63 particles were incubated with 10% normal goat serum (ZSGB-Bio, Beijing, China) for 1 h at room temperature to block non-specific binding of exosomes. Then 1mL CM was mixed with 2μL Mag-CD63 and incubated at 4°C overnight with gentle shaking. Next day, the pretreated sample was introduced into the microfluidic chip. Reagent delivery was precisely controlled at a flow rate of 2 μL/min using a double syringe programmable pump system (Baoding LongerPrecision Pump Corp., Baoding, China). PKH67 (Sigma, Saint Louis, MO,USA) was used to detect general exosome capture. MHC I (1:200; Abcam), EpCAM (1:200;R&D Systems, Minneapolis, USA), and HER 2 (1:400; Abcam) were used to detect specific exosomes. IFKine® Green Donkey Anti-Goat IgG or Dylight 549 Goat Anti-Rabbit IgG (1:500; Abbkine, Redlands, CA, USA) was used to detect exosomes expressing specific primary antibodies. Fluorescent images were observed and recorded using an inverted fluorescent microscope (Olympus IX81, Tokyo, Japan). The images were analyzed using image processing and analysis software (Image-ProPlus 6.0, Media Cybernetics, USA). The standard on-chip manipulation method was established and applied in all experiments.

### Statistical analysis

Statistical analyses were performed using SPSS version 13.0 for Windows. The correlation between Mag-CD63 concentration and captured exosomes was analyzed by Pearson correlation coefficients. Statistical significance was determined by one-way ANOVA. All experiments were repeated at least three times. Significance was defined at *p*≤ 0.05.

## Results and discussion

### A microfluidic chip for immunocapture and detection of exosomes

**Fig 1:**
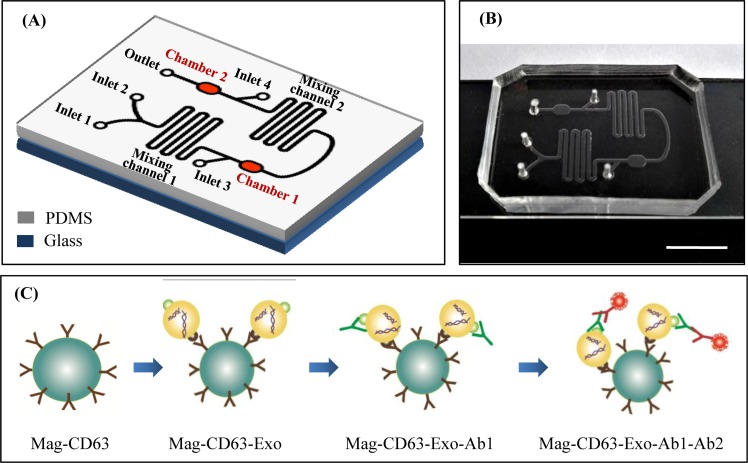
Chip design and principle for exosome capture and detection. (A) Schematic representation of the microfluidic chip. (B) Image of the chip. The scale bar represents 1 cm. (C) Workflow for the immunomagnetic capture and detection of exosomes.

The microfluidic chip consisted two chambers (100 μm height, 2500 μm width, and 3500 μm length) for immunomagnetic particle collection, two circuitous mixing channels (100 μm height, 500 μm width, and 5000 μm length), four inlets, and one outlet (Fig 1A and 1B). The principle of this study was illustrated in Fig 1C. Mag-CD63 was used to capture exosomes. The exosome-containing sample was pre-mixed with Mag-CD63 to form Mag-CD63-Exo complex. The complex was introduced into the microfluidic chip through inlet 1 and primary antibody was introduced through inlet 2. A double syringe pump drove them to flow and mix in the first mixing channel, leading to Mag-CD63-Exo-Ab1 complex formation. The immunomagnetic particles were retained in chamber 1 by a magnet disc. PBS buffer was introduced into inlet 3 and washed the Mag-CD63-Exo-Ab1 complex. Then fluorescent labeled secondary antibody was introduced through inlet 3 and driven to flow and mixed with Mag-CD63-Exo-Ab1 via the second mixing channel, then retained in chamber 2. After washed with PBS, the immunocaptured exosomes were examined by an inverted fluorescent microscope.

Due to different biomarkers expressed in exosomes from other EVs, immunocaptured exosomes present good purity with rare contamination. CD63 protein, a member of the transmembrane 4 superfamily, is one of the most abundant proteins found in exosomes and thereby commonly used for capture. Immunocapture exosomes using microfluidic technique could be achieved by two ways. The first is immobilizing antibodies on the channel surface [17, 22, 25]. To enhance the capture efficiency, microstructures could be designed in the channel providing a large surface area [25]. Compared to the microchannels, nanoparticles presented higher capture efficiency and analysis sensitivity due to the larger surface area. The magnetic nanoparticles used in our study have the size about 400 nm in diameter and presented high capture efficency. Immunomagnetic particles have been reported to capture exosomes in previous studied [23, 24]. But the magnetic particles used in these studies were larger than that used in our study with 2.8 μm diameter. Regarding the nanoparticle materials, gold may quench fluorescent signals. Similarly, we failed to detect fluorescent signals after both PKH67 labeling and immunofluorescent staining with the exosomes captured by gold-coated magnetic nanoparticles (data not shown). The shell of magnetic nanoparticles used in this study is composed of high molecular material. The expression of biomarkers on the immunocaptured exosomes could be detected by standard immunofluorescent staining. The platform developed in our study is simple to operate and especially suitable for biological researchers although it showed limited novelty.

### Characterization and quantification of the immunomagnetic captured exosomes

**Fig 2:**
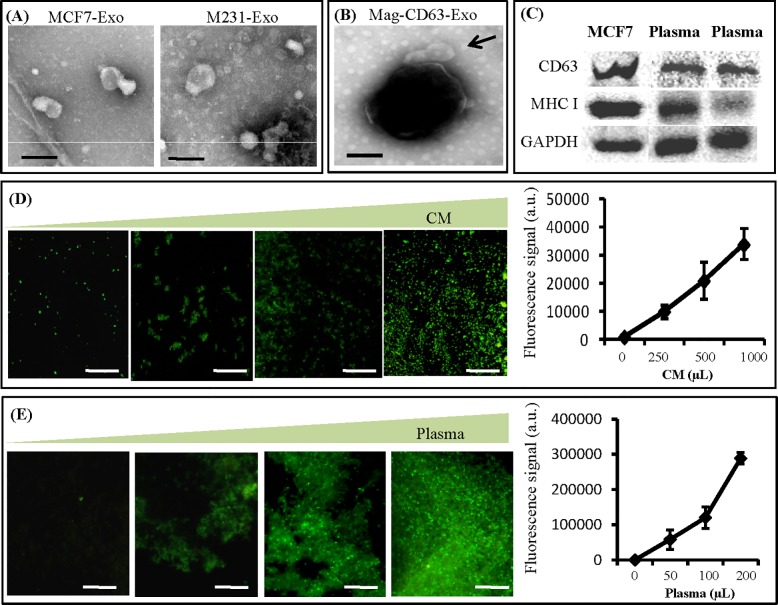
Verification of immunocapture of exosomes on the chip. (A) MCF7 and M231 secreted exosomes were negatively stained using uranyl acetate and viewed by TEM. These exosomes showed typical cup-shaped morphology with diameters less than 100 nm. (B) TEM images of the immunomagnetic particles with. The arrow indicated two exosomes captured by a magnetic nanoparticle. (C) Western blot analysis of CD63, MHC I, and GAPDH expression in exosomes isolated from MCF7 culture medium and two plasma samples. (D) Quantification of total exosomes captured by Mag-CD63 from different volumes of M231 culture medium. A stable on-chip capture efficiency was demonstrated (R^2^ = 0.93). (E) Quantification of total exosomes captured by Mag-CD63 from different volumes of human plasma sample. A stable on-chip capture efficiency was demonstrated (R^2^ = 0.995). The scale bars represent 100 nm in (A-B) and 50 μm in (D-E).

Exosomes released from MCF7 and M231 in cell culture medium were characterized by TEM. It was found that exosomes generated by both cell lines showed typical cup-shaped morphology with diameters less than 100 nm (Fig 2A). Then Mag-CD63 with or without exosome capture was examined by TEM. As shown in Fig 2B, some exosomes attached to the surface of Mag-CD63. The immunocaptured exosomes presented good integrity with similar morphology to that in Fig 2A. It suggests that Mag-CD63 could capture intact exosomes. The exosomal markers CD63 and MHC I were confirmed in exosomes purified from MCF7 culture medium and plasma samples by western blotting (Fig 2C). Stable capture efficiency is required for a new exosome manipulation technique. Studies were performed to confirm that exosomes bound to Mag-CD63 in a linear fashion. Mag-CD63 particles were mixed with different volumes of CM, respectively. The captured exosomes were stained with PKH67 and capture efficiencies were quantified (Fig 2D). It was found that the quantity of captured exosomes increased linearly with CM volume with R^2^ values of 0.93. It demonstrates that binding of exosomes to Mag-CD63 is specific and linear with the volume of CM. Similarly, it was found that binding of exosomes to Mag-CD63 is specific and linear with the volume of plasma sample (Fig 2E).

It was reported that immuno-capture of exosomes presents higher purity and less damage, compared to ultracentrifuge. In our study, captured exosomes examined by TEM showed typical features of exosomes without damage[23]. These high quality exosomes are critical for downstream analyses in both basic research and clinical practice. To visualize the captured exosomes on the microfluidic device, membrane labeling technology was applied. PKH67, a green fluorescent dye, can stably incorporate into lipid regions of the exosome membrane and provides an easy method to view and quantify the total captured exosomes. Besides intact capture, stable capture efficiency by our microfluidic device was demonstrated. It promises quantitative measurement of exosomes between different samples.

### On-chip capture and detection of exosomes from cell culture medium

**Fig 3:**
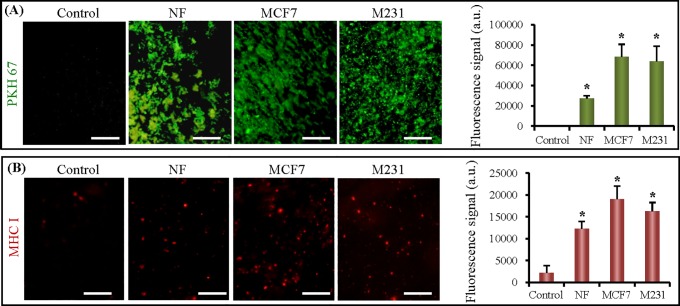
Microfluidic immunomagnetic capture and detection from cell culture medium. (A) Images and quantification of total exosomes from control and CM of NF, MCF7 and M231. Exosomes were stained by PKH67. (B) Images of and quantification of MHC I-positive exosomes from control and CM of NF, MCF7 and M231. *, *p*< 0.05. The scale bars represent 50 μm.

To verify the capability of exosome capture and detection of our method, CM of NF, MCF7, and M231 cells were analyzed on the microfluidic chip. Medium without cell culture was used as a negative control. Exosomes in each sample were on-chip measured by fluorescent labeling with PKH67. The total exosome level was quantified using the standard method established in our study. Fig 3A showed the fluorescence images of captured exosomes from control, NF, MCF7, and M231 culture medium. Significant higher fluorescence signals could be observed in NF, MCF7, and M231 groups than that in the negative control (Fig 3A). And a higher fluorescent signal was observed from cancer cell culture medium (both MCF7 and M231), compared to normal cell culture medium (NF) under the same concentration (*p*< 0.05).

The amount of MHC I-positive exosomes in NF, MCF7, and M231 culture medium was measured on-chip. A significant difference could be found between CM and negative control, suggesting that all three cells could secret MHC I-positive exosomes (Fig 3B). Although both tumor cells (MCF7 and M231) secreted more MHC I-positive exosomes than NF, no significant difference was found between them. These results demonstrated the ability of the microfluidic chip to measure specific exosomes quantitatively.

It was reported that cancer cells secret more exosomes than normal cells [11]. In this study, we found that both breast cancer cell lines secreted more exosomes than normal cells when the captured exosomes were labeled by PKH67. However, no significant difference was found in the amounts of MHC I-positive exosomes between cancer cells and normal cells even though MHC I is considered to be a commonly expressed surface marker of exosomes.We suppose that a certain number of exosomes do not express CD63 and MHC I simultaneously although both of them are exosomal general biomarkers. Thus, membrane staining is reliable to quantify the amount of total immunocaptured exosomes.

### Assistant diagnosis of breast cancer by quantification of circulating EpCAM-positive exosomes

**Fig 4:**
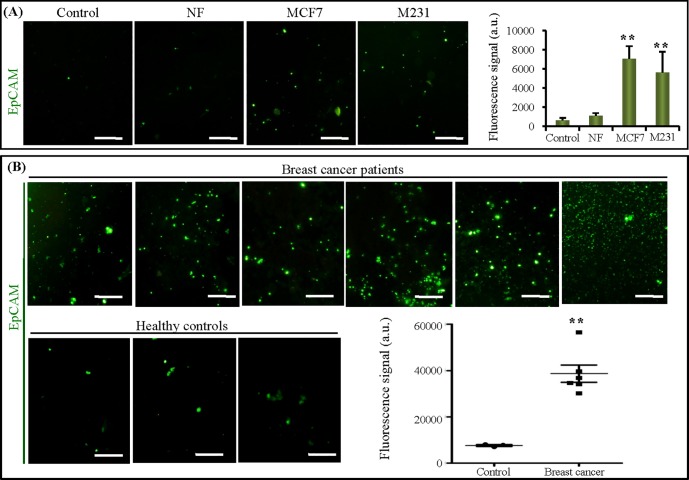
Quantification of EpCAM-positive exosomes from breast cancer cell lines and clinical patients. (A) Images and quantification of captured EpCAM-positive exosomes from Ctr, NF, MCF7 and M231 culture medium. (B) Images of and quantification of captured EpCAM-positive exosomes from plasma samples of breast cancer patients (6 cases) and healthy controls (3 cases). *, *p*< 0.05; **, *p*< 0.01. The scale bars represent 50 μm.

EpCAM is highly overexpressed in multiple types of carcinomas, including breast cancers. To assess our method in cancer diagnosis assistance, we captured EpCAM-positive exosomes from both CM and patient plasma samples using the microfluidic chip. Firstly, CM from NF, MCF7, M231 cells was on-chip analyzed, respectively. As shown in Fig 4A, the amount of EpCAM-positive exosomes from both MCF7 and M231 cell culture medium were significantly higher than that from NF culture medium and negative control (*p*< 0.01). To promote our method for clinical application, we examined circulating exosomes in clinical plasma samples collected from 6 cases of breast cancer patient and 3 cases of healthy control. Exosomes in plasma sample of each case was on-chip captured and the amount of EpCAM-positive exosomes was quantified (Fig 4B). A significant increase in the EpCAM-positive exosome level in breast cancer patients was detected, compared to healthy controls (*p*< 0.01). It suggests that the plasma exosomal EpCAM might provide diagnostic assistance for non-invasive, early detection of breast cancer.

Circulating exosomes, enriched with a group of tumor antigens, provide a unique opportunity for cancer diagnosis. EpCAM is an epithelial surface antigen (glycoprotein) found in epithelial intercellular junctions mediating homophilic calcium-dependent cell-cell adhesion [29, 30]. EpCAM frequently expressed in various solidtumors and its expression is higher in metastases of breast cancer than in the primary tumor [31, 32]. In body liquid-based diagnosis of breast cancer, EpCAM is commonly used to capture circulating tumor cells [33, 34]. The study of Kruger S et al detected EpCAM expression in M231-derived exosomes by western blot analysis [35]. However, Rupp and colleagues found that exosomes isolated from the sera of breast cancer patients lacked the EpCAM expression [36]. They supposed that it might be due to the serum contains proteolytic activity. In this study, we tried to elucidate if EpCAM could be an exosomal biomarker to assist breast cancer diagnosis. We demonstrated that on-chip captured exosomes from breast cancer patients showed different expression level of EpCAM. The average level of EpCAM-positive exosomes was significantly higher than the healthy controls. We postulate this might be due to the on-chip immunocapture method is more sensitive than traditional methods, such as western blot. Therefore, exosomes might become a new biomarkerfor personalized diagnostics in terms of their easy access and rapid response to stimuli.

### Molecular classification of breast cancer by quantification of circulating HER2-positive exosomes

**Fig 5:**
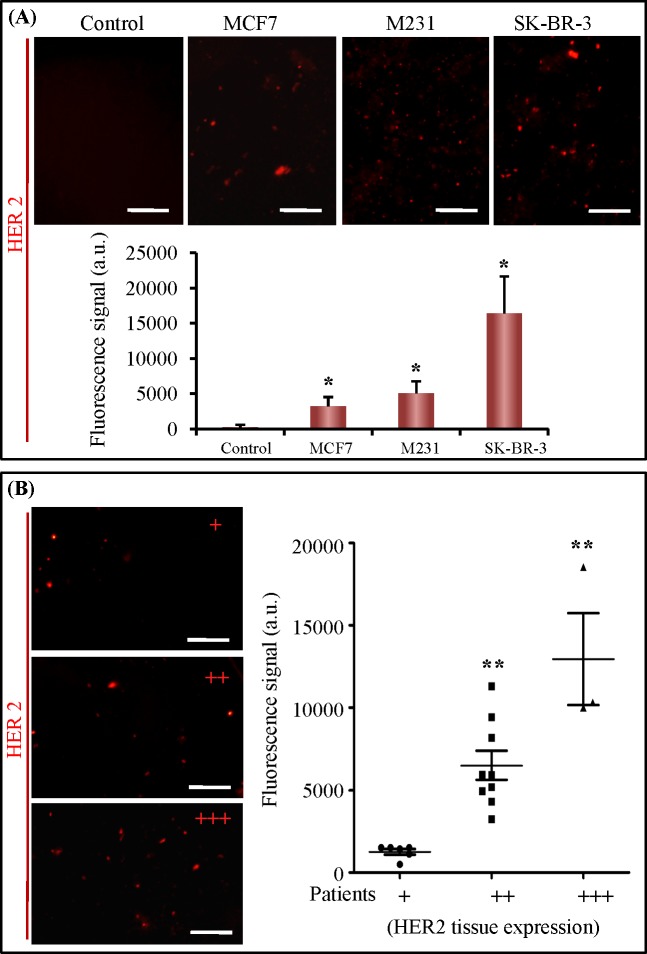
Quantification of HER2-positive exosomes from breast cancer cell lines and clinical patients. (A) Images and quantification of captured HER2-positive exosomes from Ctr, NF, MCF7, M231 and SK-BR-3 culture medium. (B) Images and quantification of captured HER2-positive exosomes from plasma samples of breast cancer patients with different HER2 expression in primary tumor tissues. HER2+, 6 cases; HER2++, 9 cases; HER2 +++, 3 cases.*, *p*< 0.05; **, *p*< 0.01. The scale bars represent 50 μm.

Overexpression of HER2 is responsible for malignant transformation of mammary epithelial cells, with approximately 25% of invasive primary breast tumors exhibiting HER2 gene amplification. Trastuzumab, a monoclonal antibody targeting HER2, is widely used as an approved treatment for advanced metastatic and early disease. Thus molecular classification by HER2 is very important in personalized therapy for breast cancer patients. A recent study revealed that HER2-positive exosomes play an important role in modulating sensitivity to Trastuzumab. To investigate the amount of HER2-positive exosomes secreted by breast cancer cells, we on-chip analyzed CM from MCF7, M231, SK-BR-3 cells and detected exosomes with monoclonal antibodies specific for HER2. Our on-chip results showed that HER2-positive exosomes could be detected in all of CM, but not in negative control (Fig 5A). There was a significant difference between them. Furthermore, HER2-positive exosome level showed significantly higher in SK-BR-3 CM than in both MCF7 and M231 CM. This result was consistent with previous study that both MCF7 and M231 showed low HER2 expression and SK-BR-3 cells expressed high level HER2.

We further investigated the level of circulating HER2-positive exosomes in 19 cases of breast cancer patient using the microfluidic chip. HER2 expression level of each case was determined in clinic by immunohistochemical staining using tumor tissues. Clinical analysis showed that 5 cases were HER2 negative expression (+), 11 cases were HER2 moderate expression (++), and 3 cases were HER2 strong expression (+++). Our on-chip analysis results were shown in Fig 5B. The average level of HER2-positive exosomes showed increasing tendency from HER2 negative to moderate and strong tissue expression cases. Although the exosomal HER2 expression were different in 9 cases with HER2 moderate tissue expression, their average exosomal HER2 was significantly higher than that with HER2 negative tissue expression. Similarly, the average exosomal HER2 expression in HER2 strong tissue expression cases was significantly higher than that with HER2 moderate and negative tissue expression cases.

The practice of ‘‘one medicine for all patients with the same diseases” cannot stand any more. Personalized medicine suggests that the tumor therapy should be determined according to individual characteristics and responses to the specific treatment [15, 37]. The development of trastuzumab has dramatically increased the survival in HER2-positive metastatic breast cancer [38–40]. Thus, HER2 testing is important for an effective anticancer treatment strategy. As exosomes carry various molecular components of their cell of origin, analysis for the surface HER2 expression of exosomes in serum might provide an alternative method to diagnose HER2 status in breast cancer patients [27, 41].Our study demonstrated that the amount of circulating HER2-positive exosomes was almost parallel with HER2 expression in primary tumor tissues. Measuring exosomal HER2 expression using the microfluidic chip required small volume, low cost, and shorter time. Therefore, this new platform provides an alternative method for molecular classification of breast cancer patients [42].

## Conclusions

As exosomes have the potential to be a kind of liquid biopsy with a higher sensitivity and accuracy, developing new exosome capture and detection platform is of great significance. In this study, we have developed a microfluidic device that enables immunocapture and quantification exosomes from both cell culture medium and patient’s serum. Immunocapture of exosomes avoids contamination of other EVs and mechanical damage, leading to higher purity and intact yield. Taking the advantages of microfluidic technique, small volume sample is required for analysis. And the operation is simple, time-saving, and low cost. Clinical application proved that the microfluidic device should provide a tool to assist breast cancer diagnosis and molecular classification for personalized treatment.

## Supporting information

S1 File Microfluidic-based liquid biopsy to assist cancer diagnosis. (DOCX)
