# Supplementary material for: Clinical application of a microfluidic chip for immunocapture and quantification of circulating exosomes to assist breast cancer diagnosis and molecular classification
Source: PLoS One. 2017 Apr 3;12(4):e0175050. doi: 10.1371/journal.pone.0175050 (PMC5378374; doi:10.1371/journal.pone.0175050)
Supplement: S1 File — (DOCX) [file pone.0175050.s001.docx]

Caption of graphic abstract:

Microfluidic-based liquid biopsy to assist cancer diagnosis
